# Supplementary material for: Saccharomyces boulardii (CNCM I-745) improves intestinal damage in sepsis by remodeling intestinal flora structure
Source: Front Cell Infect Microbiol. 2025 Jul 31;15:1602792. doi: 10.3389/fcimb.2025.1602792 (PMC12350329; doi:10.3389/fcimb.2025.1602792)
Supplement: Supplementary file 1 [file DataSheet1.zip › summary/summary/8_Advanced_analyse/2_PICRUSt2_result/METACYC/Group.DW0_vs_CW0_vs_BW0_vs_AW0/PICRUSt2_METACYC_Group.DW0_vs_CW0_vs_BW0_vs_AW0_diff.pdf]

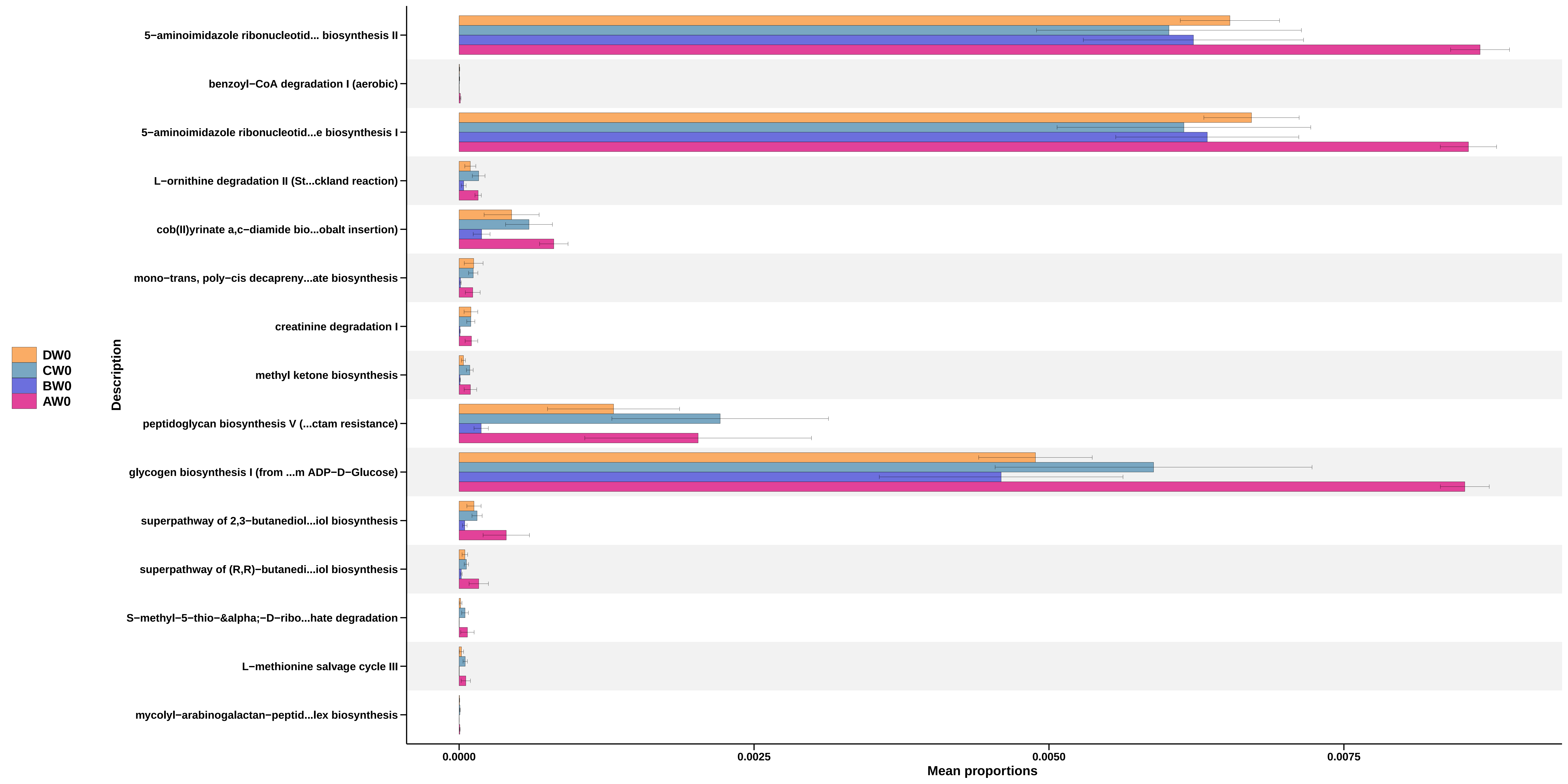
5−aminoimidazole ribonucleotid... biosynthesis II
benzoyl−CoA degradation I (aerobic)
5−aminoimidazole ribonucleotid...e biosynthesis I
L−ornithine degradation II (St...ckland reaction)
cob(II)yrinate a,c−diamide bio...obalt insertion)
mono−trans, poly−cis decapreny...ate biosynthesis
creatinine degradation I
methyl ketone biosynthesis
peptidoglycan biosynthesis V (...ctam resistance)
glycogen biosynthesis I (from ...m ADP−D−Glucose)
superpathway of 2,3−butanediol...iol biosynthesis
superpathway of (R,R)−butanedi...iol biosynthesis
S−methyl−5−thio−α−D−ribo...hate degradation
L−methionine salvage cycle III
mycolyl−arabinogalactan−peptid...lex biosynthesis
Description
DW0
CW0
BW0
AW0
0.0000
0.0025
0.0050
0.0075
Mean proportions
